# mHealth App Usability Questionnaire for Stand-Alone mHealth Apps Used by Health Care Providers: Canadian French Translation, Cross-Cultural Adaptation, and Validation (Part 1)

**DOI:** 10.2196/50839

**Published:** 2024-02-13

**Authors:** Julie Gagnon, Sebastian Probst, Julie Chartrand, Michelle Lalonde

**Affiliations:** 1 School of Nursing Faculty of Health Sciences University of Ottawa Ottawa, ON Canada; 2 Département des sciences de la santé Université du Québec à Rimouski Rimouski, QC Canada; 3 Haute École Spécialisée de Suisse occidentale University of Applied Sciences and Arts Western Switzerland Geneva Switzerland; 4 Care Directorate University Hospital Geneva Geneva Switzerland; 5 College of Medicine Nursing and Health Sciences University of Galway Galway Ireland; 6 Children’s Hospital of Eastern Ontario Research Institute Ottawa, ON Canada; 7 Institut du Savoir Montfort Montfort Hospital Ottawa, ON Canada

**Keywords:** cross-cultural adaptation, French language, mHealth App Usability Questionnaire, MAUQ, mobile health, mHealth, mobile app, questionnaire translation, usability, validation, health care providers, French translation

## Abstract

**Background:**

An increasing number of health care professionals are using mobile apps. The mHealth App Usability Questionnaire (MAUQ) was designed to evaluate the usability of mobile health apps by patients and providers. However, this questionnaire is not available in French.

**Objective:**

This study aims to translate (from English to Canadian French), cross-culturally adapt, and initiate the validation of the original version of MAUQ for stand-alone mobile health apps used by French-speaking health care providers.

**Methods:**

A cross-cultural research study using a well-established method was conducted to translate MAUQ to Canadian French by certified translators and subsequently review it with a translation committee. It was then back translated to English. The back translations were compared with the original by the members of the committee to reach consensus regarding the prefinal version. A pilot test of the prefinal version was conducted with a sample of 49 potential users and 10 experts for content validation.

**Results:**

The statements are considered clear, with interrater agreement of 99.14% among potential users and 90% among experts. Of 21 statements, 5 (24%) did not exceed the 80% interrater agreement of the experts regarding clarity. Following the revisions, interrater agreement exceeded 80%. The content validity index of the items varied from 0.90 to 1, and the overall content validity index was 0.981. Individual Fleiss multirater κ of each item was between 0.89 and 1, showing excellent agreement and increasing confidence in the questionnaire’s content validity.

**Conclusions:**

This process of translation and cultural adaptation produced a new version of MAUQ that was validated for later use among the Canadian French–speaking population. An upcoming separate study will investigate the psychometric properties of the adapted questionnaire*.*

## Introduction

### Background

Mobile health (mHealth) is increasingly used in health care systems, and this fast-growing technology has immeasurable potential to improve the quality and accessibility of health care worldwide [1]. There is no exact figure for the number of mHealth apps available worldwide, as this number is constantly changing owing to the launch of new apps and the removal of existing ones. Globally, the number of mHealth apps for patients and health care providers exceeded 350,000 in 2021 [2]. For care providers, mHealth apps are a fast and effective way to improve communication between patients and interdisciplinary teams. They also enable more accurate data collection at patients’ bedside, facilitate documentation, and increase the availability of care for people living in rural or remote areas [1]. The effectiveness and efficacy of mHealth apps must be guaranteed to optimize the use of this limitless resource, improve user experience, and benefit from the subsequent reduction in health care system costs [3].

Ensuring the usability of mHealth apps is an important step in their development and evaluation. However, literature indicates a lack of evidence regarding the quality of mHealth apps and no legal framework at the national policy level [4,5]. In mHealth, usability refers to the ease and efficiency with which users will use a tool to satisfactorily accomplish a specific task [6]. This includes aspects such as ease of use, operability, clarity of instructions, risk of errors and possibility of correcting them, and user-friendliness of the interface [7].

Currently, several questionnaires are available for evaluating mobile apps. The Mobile Application Rating Scale (MARS) and the user version of MARS for evaluating the quality of mHealth apps in the broadest sense are among the most widely used measures [8,9]. A systematic review, including 87 studies published between 2000 and 2018 [10], highlighted that the usability scales used to evaluate mHealth apps were all initially created to obtain the perspective of developers and researchers. Questionnaires for assessing the usability of mHealth apps by different users have since been created but not yet validated. One of these questionnaires is the multidimensional App Quality Assessment Tool for Health-Related Apps that can be used by experts and users to quickly determine the quality of health-related and mental health–related apps [11]. The mHealth App Usability Questionnaire (MAUQ) is the only questionnaire specifically validated for stand-alone mHealth apps used by health care providers [12]. It is originally available in English, and MAUQ for stand-alone mHealth apps for patients was translated to Malay and validated by a Malaysian research team [13].

### The Need for a Canadian French Questionnaire

It is well known that cultural differences can influence how participants respond to questions associated to the measurement tools owing to dissimilarities in language and social and professional norms [14]. Therefore, cultural bias can creep into study results and influence their interpretation [15]. Ensuring the translation, cross-cultural adaptation, and validation of measurement tools beforehand is a recognized process for minimizing this bias and ensuring the validity of study results [16,17].

With 321 million speakers, French is the fifth most spoken language in the world [18]. As a member state of Francophonie, Canada has a vast territory that is rich in linguistic diversity. Spanning 5514 km between the Pacific and Atlantic oceans, Canada has 2 official languages: English and French. The proportion of Canadians with French as their mother tongue is 20.9% [19], and the number of French-speaking researchers is 63,455 [20]. Although both languages are spoken across the country, French remains as the majority language in the province of Quebec, which accounts for 85.5% of Canadians with French as their mother tongue [19]. Francophone Canadian researchers are also interested in the contribution of mobile technologies to health but have access to very few reliable and valid instruments in French. Clearly, the lack of valid measurement tools in French affects the ability to study this population [20]. This puts francophone health care providers at a disadvantage, as they are often left out of studies available exclusively to anglophone participants. Thus, their experiences are less represented in literature [21].

Currently, there are only few measurement tools available in French such as MARS [22] or Unified Theory of Acceptance and Use of Technology 2 [23,24]. So far, there is no French version of MAUQ. Consequently, there is a necessity for a measurement tool that is translated, cross-culturally adapted, and validated for use with Canadian French health care providers.

This study was the first of 2 phases of a methodological study. The aims of the first phase were the Canadian French translation and cross-cultural adaptation of MAUQ and the initiation of its validation to allow Canadian French health care providers to eventually evaluate the usability of mHealth apps.

## Methods

This paper has described the Canadian French translation, cross-cultural adaptation, and validation of the original version of MAUQ. The second step in the assessment of the psychometric properties of the translated version will be described in a later publication.

### Instrument

The original version of MAUQ was developed to quantitatively measure the usability of mHealth apps by patients and health care providers regarding ease of use, interface design, user satisfaction, and usefulness, before their launch to the general public [12]. Originally in English, MAUQ was created and validated by health informatics professor Leming Zhou and his colleagues at the University of Pittsburgh [12]. The authors point out that there are no licensing fees for using the questionnaire, and it is not necessary to request permission before using it. The questionnaire is freely accessible on the website [25] and is available in 4 versions, according to app type (interactive or stand-alone) and target population (patients or health care providers). This study was conducted using MAUQ for stand-alone mHealth apps used by health care providers.

MAUQ for stand-alone mHealth apps used by health care providers consists of a short guideline for completing the questionnaire, followed by 18 statements and an open question for comments. The statements address 3 domains: ease of use (questions 1-5), interface and satisfaction (questions 6-12), and usefulness of the mobile app (questions 13-18). The statements were developed based on a systematic literature review of 312 unique questionnaire statements from 38 questionnaires. People completing MAUQ are asked to rate their level of agreement on a Likert scale ranging from 1 (disagree) to 7 (agree). App usability is determined by the total average of all scored items for each participant: the higher the average, the better the app’s usability. It is also possible to evaluate the responses to each item to assess a specific component of usability and compare the averages.

The validity study conducted by MAUQ authors used only the 2 patient versions [12]. The authors report that the differences between the patient and health care provider versions are negligible. Initially conducted with 128 participants from the University of Pittsburgh’s academic community, the validity study of MAUQ designed for stand-alone mHealth apps demonstrated strong internal reliability, with an overall Cronbach α value of .914 for the entire questionnaire and .847, .908, and .717 for ease of use, interface and satisfaction, and usefulness, respectively [12].

### Translation, Adaptation, and Validation Processes

The accepted method of instrument translation and cultural adaptation suggested by Sousa and Rojjanasrirat [17] was retained for this study (Table 1). This 7-step sequential method incorporates the recommendations of the most established methodological approaches in a clear and detailed guideline. Moreover, it aims to provide a symmetrical translation, which is the most recommended because it remains true to the intended meaning and linguistic expression in equal measure between the 2 languages (that of the source instrument and the target instrument) [17,26,27]. Ultimately, the objective of this method was to achieve equivalence between the original and translated versions of the questionnaire. The cross-cultural equivalence is broken down by Flaherty et al [28] into 5 mutually exclusive equivalences of semantic, technical, conceptual, content, and criterion origin (defined in Textbox 1).

**Table 1:** The 7-step guideline for translation, cross-cultural adaptation, and validation according to Sousa and Rojjanasrirat [17] and the respective equivalences achieved.

Steps			Equivalences								
			Content		Semantic		Technical		Criterion		Conceptual
**Completed in full in this study**											
	Obtaining authorization to translate the tool; independent, double translation from English to French			✓							
	Comparison of the 2 translated versions, discussion, and consensus regarding a preliminary French version			✓							
	Independent double back translation from French to English			✓							
	Comparison of the 2 back-translated versions with the original; discussion and consensus regarding the prefinal French version	✓		✓						✓	
	Pilot study to test the prefinal French version	✓		✓		✓				✓	
**Separate studies (upcoming)**											
	Preliminary psychometric test with a bilingual sample (French-English)	✓		✓		✓		✓		✓	
	Complete psychometric test	✓		✓		✓		✓		✓

**Definitions of the equivalences to be achieved in the cross-cultural validation process according to Flaherty et al [28].**

**Content**

- The content of each questionnaire statement is relevant to each culture.

**Semantic**

- The meaning of each statement is the same in each culture after translation.

**Technical**

- The data collection method (in this case, a questionnaire) is comparable in each culture in terms of the data it reports.

**Criterion**

- The interpretation of the measurement of each variable and of the results is the same when compared between the 2 cultures.

**Conceptual**

- The theoretical constructs evaluated and the concepts used are the same in both cultures.

### Step 1

Step 1 consisted of the independent and anonymous forward translation of the original MAUQ from English to French by 2 professional translators with French as their mother tongue. One of the 2 translators was familiar with digital health terminology, and the other was familiar with the cultural and linguistic nuances of French.

### Step 2

In step 2, a comparison of the 2 translated versions with the original was performed by a team comprising the 2 translators, a nurse (JG; member of the research team), and a third-party translator to assess the degree of equivalence of the translation. Ambiguities and differences between words, phrases, grammar, and meanings were discussed in a virtual meeting to reach consensus regarding the first version of the translated MAUQ.

### Step 3

Step 3 involved the independent and anonymous back translation of the translated version to English by 2 other certified translators with English as their mother tongue and no previous knowledge about the original MAUQ. They had to consider the French version as the original.

### Step 4

In step 4, a committee (n=6) compared these 2 back-translated versions with the original MAUQ version to assess the degree of equivalence of the back translations. This committee, which included all 4 bilingual and bicultural translators who worked in steps 1 and 3, a nurse (JG; member of the research team), and a health care provider (an experienced acute care nurse), was formed to discuss ambiguities and differences between words, phrases, grammar, and meanings. Consensus for each of the statements was established during a virtual meeting, ensuring consistency and clarity of formulation according to the Canadian French language and culture. The prefinal version of the translated and adapted questionnaire was consolidated and named MAUQ en français (MAUQ-FR).

At each of these first 4 steps, 1 of the 2 certified translators was familiar with digital health terminology, ensuring that the constructs of the tool were understood. All the involved individuals were bilingual experts.

### Step 5

Step 5 consisted of pilot-testing MAUQ-FR with target users and a panel of unilingual experts. For the target population, Sousa and Rojjanasrirat [17] define participants as people whose language is the target language of the instrument and who should be recruited from the target population in which the instrument will be used. A group of 49 registered nurses with French as their mother tongue completed a 5-minute SurveyMonkey (Symphony Technology Group) questionnaire asking them to rate the clarity of the instructions and each translated MAUQ statement dichotomously (*clear* or *unclear*) [17,29]. If they selected *unclear*, a textbox appeared, so that they could indicate how to rewrite the statement to make it clear. Recruitment with voluntary sampling was conducted among graduate nurses from a Quebec university.

The same approach was used with an expert panel, in addition to rating the relevance of each statement regarding their experience with Canadian health care. To achieve this, a Likert scale ranging from 1 (not relevant) to 4 (very relevant) was used to avoid a neutral position [30,31]. Sousa and Rojjanasrirat [17] indicate that the panel should consist of experts “who are knowledgeable about the content areas of the construct of the instrument and the target population in which the instrument will be used and whose mother language is the target language of the instrument.” A search was conducted across Canada to find experts who are using mobile technology at work and with French as their mother tongue. Following the target number of 6 to 10 experts [30,32], the 10 people who assessed content validity were 2 (20%) professors in nursing, 1 (10%) person in public health who works on the evaluation of information and communication technologies and its specific terminology, 3 (30%) doctoral candidates and professors in nursing, 1 (10%) physician and clinical professor in medicine, 1 (10%) mobile app developer, and 2 (20%) health-related practitioners who use mHealth (1 nurse manager and 1 medical specialist). Experts were recruited from the Canadian provinces of Manitoba, Ontario, Quebec, and New Brunswick through networking (colleagues and contacts). Participation in this study was entirely voluntary.

As recommended by Sousa and Rojjanasrirat [17], this first study only covered steps 1 to 5. Steps 6 and 7 involving the evaluation of the psychometric properties (Cronbach α) and the measurement of the internal consistency reliability (Lin concordance correlation coefficient) of MAUQ-FR with a bilingual, French-English sample (target n=90) and the target population (target n=180) will be conducted in 2 subsequent studies.

### Analyses for the Validation of the Instrument

The quantitative data obtained during the pilot test were extracted directly from the SurveyMonkey website and analyzed using descriptive statistics presented as frequencies and percentages, including interrater agreement. The minimum interrater agreement was set at 80% [17]. The research team revised and reevaluated the statements rated as *unclear* by at least 20% (2/10) of the sample, in addition to considering all feedback obtained from *unclear* responses to improve MAUQ-FR.

Data collected from the expert panel made it possible to assess content validity with the content validity index (CVI): CVI at item level (I-CVI) and CVI at scale level (S-CVI). Relevance scores were previously dichotomized: scores of 1 and 2 were coded as 0 (not relevant) and scores of 3 and 4 were coded as 1 (relevant) [30]. With 10 experts, the minimum thresholds to reach were at least 0.79 for I-CVI [30] and at least 0.80 for the averaging calculation at S-CVI [32,33]. Considered as the average of the proportion of items deemed relevant across the various judges, S-CVI was calculated by adding I-CVIs and dividing by the number of items [33].

Members of the research team considered and discussed the statements with a relevance score of 1 (not relevant) or 2 (unable to assess relevance). Items that failed to meet the previously indicated I-CVI thresholds were revised and reevaluated by the expert panel. New validity indices were then calculated until acceptable I-CVIs were reached. The modified κ coefficient of agreement (Fleiss multirater κ) was also calculated to determine interrater agreement among experts [34,35]. A κ of 0.60 is considered as the minimum acceptable coefficient to determine good agreement, whereas a value ≥0.75 is considered as excellent [34,36]. All statistical analyses were performed using Microsoft Excel.

### Ethical Considerations

After submission to the research ethics board at University of Ottawa, an approval from the research ethics board will be required only for subsequent stages (psychometric testing), since this study is regarded as a quality improvement study. All participants received the information about the objectives of the study, procedures involved, and confidentiality of the data. Informed consent was obtained from all participants. In accordance with the chosen methodology, the completed questionnaires were entirely anonymous and did not collect sociodemographic data. Authorization to translate MAUQ was obtained in advance from the authors.

## Results

### Steps 1 to 4: Translation

Steps 1 to 4 helped to achieve conceptual, semantic, and content equivalence. The translated version includes the 3 domains of the original version of MAUQ, which have been similarly broken down into 18 statements. In more detail, the step-2 consensus phase made it possible to work on semantic equivalence, ensuring that there was no change in the meaning of the words used in the original questionnaire. The committee met virtually for 1 hour. As there was hesitation in choosing the right terms, the translators were encouraged to indicate all possible options for certain words to clarify their connotations and jointly make the best decision (eg, user-friendliness vs use vs usability).

The step-3, independent, double back translation clarified the words and sentences used in the translation to determine the accuracy of the translation by identifying the differences between the 2 English versions (semantic equivalence). In a 2-hour virtual meeting, the step-4 committee discussion validated each statement and established conceptual, semantic, and content equivalences. Professor Zhou, author of the original questionnaire [12], was contacted to clarify the intended meaning of the term “social settings” in the ninth statement. Then, 4 statements were modified between the step-2 and step-4 consensuses (Table 2). Following these modifications, the committee unanimously reached consensus that the words and concepts used complied with the language and each cultural perspective.

**Table 2:** Grammatical changes between the step-2 and step-4 consensuses of the translation and cross-cultural adaptation process.

Item number	Step-2 statement	Step-4 statement
9	“Je suis à l’aise d’utiliser l’application dans un contexte de soins communautaires” (I am comfortable using the application in a community care setting)	“Je suis à l’aise d’utiliser l’application dans un endroit public” (I am comfortable using the application in a public place)
10	“temps nécessaire” (time needed)	“temps requis” (time required)
Items 14 and 18	“services de soins de santé” (health-care services)	“services de soins” (care services)

### Step 5: Pilot Test (Target Population)

For face validation, the French-speaking registered nurses (n=49) considered the statements to be clear, with interrater agreement of 99.14% (Table 3). In total, 5 comments were collected and considered to improve the questionnaire. This pilot test provided additional support for conceptual equivalence (clarity) and content equivalence (relevance) in the Canadian French cultural context.

**Table 3:** Interrater agreement on statement clarity among the target population and the expert panel during the pilot test.

Statement	Target population (n=49), n (%)^a^	Round-1 experts (n=10), n (%)^b^	Round-2 experts (n=9), n (%)^c^
Title	45 (92)	9 (90)	N/A^d^
Directives	49 (100)	10 (100)	N/A
Item 1	48 (98)	10 (100)	N/A
Item 2	49 (100)	10 (100)	N/A
Item 3	46 (94)	8 (80)	9 (100)
Item 4	49 (100)	9 (90)	N/A
Item 5	49 (100)	10 (100)	N/A
Item 6	49 (100)	9 (90)	N/A
Item 7	49 (100)	9 (90)	N/A
Item 8	49 (100)	8 (80)	9 (100)
Item 9	49 (100)	9 (90)	N/A
Item 10	49 (100)	10 (100)	N/A
Item 11	49 (100)	9 (90)	N/A
Item 12	49 (100)	9 (90)	N/A
Item 13	49 (100)	10 (100)	N/A
Item 14	49 (100)	8 (80)	9 (100)
Item 15	49 (100)	9 (90)	N/A
Item 16	48 (98)	9 (90)	N/A
Item 17	49 (100)	10 (100)	N/A
Item 18	49 (100)	8 (80)	8 (89)
Conclusion	49 (100)	6 (60)	9 (100)

^a^Interrater agreement within the target population=99.14.

^b^Interrater agreement among round-1 experts=90.

^c^Interrater agreement among round-2 experts=93.

^d^N/A: not applicable.

### Step 5: Expert Panel

The experts (n=10) considered the statements to be clear, with interrater agreement of 90% (Table 3). The 5 statements that did not exceed 80% interrater agreement were revised by the research team and reevaluated by the expert panel (9/10, 90%) to achieve content-related validity. Interrater agreement for the modified statements was 93%.

I-CVI for each statement ranged from 0.90 to 1, and S-CVI was 0.981 (Table 4). Individual Fleiss multirater κ for each item ranged from 0.89 to 1, increasing confidence in the questionnaire’s content validity [35]. There were 32 comments, which improved the accuracy of the statements.

Step 5 helped to reinforce the conceptual, semantic, and content equivalence and prepare a translated and adapted version. The sample sizes required were achieved and even exceeded for the pilot test with the target population.

In short, all the items and the title, instructions, and conclusion met the thresholds for psychometric testing. The example in Table 5 illustrates the entire process of steps 1 to 5.

**Table 4:** Content validity index (CVI) of item relevancy and Fleiss κ agreement by the expert panel during the pilot test.

Statement	Rating of 1 or 2 (n=10), n (%)	Rating of 3 or 4 (n=10), n (%)	Item-level CVI^a^	Fleiss κ	Interpretation
Title	0 (0)	10 (100)	1.00	1.00	Excellent
Directives	0 (0)	10 (100)	1.00	1.00	Excellent
Item 1	0 (0)	10 (100)	1.00	1.00	Excellent
Item 2	0 (0)	10 (100)	1.00	1.00	Excellent
Item 3	1 (10)	9 (90)	0.90	0.89	Excellent
Item 4	0 (0)	10 (100)	1.00	1.00	Excellent
Item 5	0 (0)	10 (100)	1.00	1.00	Excellent
Item 6	0 (0)	10 (100)	1.00	1.00	Excellent
Item 7	0 (0)	10 (100)	1.00	1.00	Excellent
Item 8	1 (10)	9 (90)	0.90	0.89	Excellent
Item 9	1 (10)	9 (90)	0.90	0.89	Excellent
Item 10	0 (0)	10 (100)	1.00	1.00	Excellent
Item 11	0 (0)	10 (100)	1.00	1.00	Excellent
Item 12	0 (0)	10 (100)	1.00	1.00	Excellent
Item 13	0 (0)	10 (100)	1.00	1.00	Excellent
Item 14	0 (0)	10 (100)	1.00	1.00	Excellent
Item 15	0 (0)	10 (100)	1.00	1.00	Excellent
Item 16	0 (0)	10 (100)	1.00	1.00	Excellent
Item 17	0 (0)	10 (100)	1.00	1.00	Excellent
Item 18	0 (0)	10 (100)	1.00	1.00	Excellent
Conclusion	1 (10)	9 (90)	0.90	0.89	Excellent

^a^Overall scale CVI=0.981.

**Table 5:** Translation process: example (item 16).

Original English version	Forward translation (English to French)	Consensus	Back translation (French to English)	Consensus	Pilot test
This app has all the functions and capabilities I expected it to have.	Translator 1: L’application possède toutes les fonctions et capacités auxquelles je m’attendais. Translator 2: Cette application comporte toutes les fonctions et capacités auxquelles je m’attendais.	L’application comportait toutes les fonctions et capacités auxquelles je m’attendais.	Translator 3: The app had all the features and functions I was expecting. Translator 4: The app had all of the features and capabilities I was expecting.	L’application comportait toutes les fonctions et capacités auxquelles je m’attendais.	L’application comportait toutes les fonctionnalités auxquelles je m’attendais.

## Discussion

### Principal Findings

This study made it possible to translate, cross-culturally adapt, and initiate the validation of the original, English MAUQ in Canadian French. Study results indicate that MAUQ-FR has high content validity. CVI is high for all individual items (>0.90) and for the overall scale (0.981), exceeding the minimum thresholds of 0.79 and 0.80, respectively [30,32]. These results are comparable with I-CVIs of the version translated to Malay, which varied between 0.9 and 1, and the overall S-CVI of 0.983 [13]. It is important to distinguish between item-level (I-CVI) and scale-level (S-CVI) content validity as it helps to identify specific elements of the scale that do not effectively measure the desired construct. The κ statistic showed excellent interexpert agreement. These results suggest that MAUQ-FR has been accurately translated and adapted for future francophone users in Canada.

A renowned, systematic method was used to ensure linguistic and cultural equivalence [28,29,37]. The guideline by Sousa and Rojjanasrirat [17] for achieving the objectives provided a clear and precise approach. Translation and cross-cultural adaptation studies must follow a rigorous process, as instruments simply translated from one language to another may lose their validity and no longer measure what they intended to measure, in addition to jeopardizing safety and research ethics [38]. As Sperber [39] points out in his methodological paper, the translation, cross-cultural adaptation, and validation processes are often treated as afterthoughts in research protocols. Forward translation by uncertified translators is also a commonly used methodological approach [17]. Nevertheless, it is not only essential to translate words in the literary sense but they must, most importantly, also be closely related to the context [40]. This premise is especially critical here, considering that idiomatic expressions vary in each French-speaking region of the vast Canadian territory. Being aware of possible variations involving colloquialisms and jargon, the certified translators, consensus committee members, and experts involved in this study sought to use the most common and neutral vocabulary possible, while paying close attention to cultural nuances (hence the importance of involving translators who come from both cultures or who are bicultural). Beck et al [41] used the same qualitative approach for their cross-cultural study, in which the authors highlighted the need to go beyond the search for equivalence in the denotative meaning of words. Rather, there is a great need to grasp their meaning and connotation within the cultural context they are used. All things considered, the approach was a success, and the results were validated by the group of experts from different French-speaking regions of Canada.

Despite the methodological process, the adaptation of measuring instruments between 2 cultures rarely results in perfect transposition [28]. Some equivalences are more strongly achieved than others. In this study, combining the expertise of translators, researchers, IT specialists, and health care providers favorably contributed to the thorough evaluation of semantic and conceptual equivalences [42]. Choosing qualified and certified translators and having a second independent team for back translation enabled the development of a high-quality instrument by minimizing idiosyncratic bias. Moreover, back translation has long been recognized as a key method for achieving semantic equivalence by ensuring that the translation matches the characteristics of the original instrument [26]. It also allowed the research team to verify the quality of the translation by comparing the 2 English versions of MAUQ (the original and retranslated versions). Few errors were found, attesting to the quality of the previously completed translation and consensus work.

The sequential form of the study allowed for the progressive improvement of MAUQ-FR by identifying ambiguities or terminological imprecision that had not been raised by the translation team. For example, the translation of the item, “The navigation was consistent when moving between screens,” did not exceed the 80% threshold of agreement between the experts, highlighting a lack of clarity in the translated version. Corrections were made by the research team based, among other things, on the feedback received. I-CVI of the revised version of this item finally reached 100%, ensuring conceptual equivalence. An essential element in the process was the outstanding collaboration with the principal author of the original questionnaire, which enabled fluid communication and clarification of the original meaning of certain items. Finally, the equivalences were deemed to have been satisfactorily achieved, making it possible to proceed to the evaluation of the psychometric properties of MAUQ-FR.

Once the cross-cultural validation process is completed, it will be possible to use MAUQ-FR in a comparable way in different cultures while ensuring data comparability. This will ultimately make it possible to distinguish significant differences between cultures. MAUQ-FR will enable even unilingual anglophone researchers to collect data from francophone Canadians, a population that is currently understudied [20]. Given that French is the world’s fifth most spoken language [18], MAUQ’s French translation can help to create opportunities for other cultural adaptations.

### Limitations

This study has its limitations. First, the sociodemographic data of the participants were not collected, as they are not required by the chosen method [17]. However, this prevents certain factors from being considered during the validation process, such as professional experience, age, sex, and gender.

Another limitation is that the pilot test in the target population was conducted exclusively by nurses, whereas the questionnaire could be used by other health care providers. This excluded other potential participants, such as physicians, physiotherapists, respiratory therapists, and other health care providers. In addition, the target population sample was drawn from a university in Quebec (Canada), the province with the largest number of French speakers in the country [19]. These 2 constraints make it impossible to generalize the results to all French-Canadian health care providers. The same applies to the experts surveyed. Although they come from different Canadian provinces, it would be essential to eventually include participants from other French-speaking minority regions such as the Yukon Territory and British Columbia [19]. In addition, the recruitment of experts through networking may have induced a selection bias within the panel. The participants selected were nonetheless representative of the majority of mHealth app users and able to provide a reliable evaluation of the questionnaire.

Although the pilot test allowed for the assessment of conceptual equivalence, question comprehension, and content validity, it does not guarantee construct validity, internal consistency reliability, or fidelity [17,29]. Additional studies must be conducted with full psychometric testing of a large sample of health care providers to establish Cronbach α, internal consistency reliability (Lin concordance correlation coefficient), stability reliability (test-retest), homogeneity, construct-related validity with scale and item analysis, Pearson correlations, and exploratory and confirmatory factor analysis.

### Conclusions

In summary, this study is based on the domains of equivalence by Flaherty et al [28] and was conducted in accordance with the methodology by Sousa and Rojjanasrirat [17] to achieve the translation from English to Canadian French, cross-cultural adaptation, and initiation of the validation of MAUQ. Initial tests performed with MAUQ-FR show excellent validity. As part of a doctoral research project, this adaptation was necessary to meet the specific circumstances of the population to be studied, and to ensure the methodological rigor of future studies. Finally, this study was the first phase of a methodological study and will enable the continuation of work with the psychometric evaluation of MAUQ-FR. The data collected will be shared with the authors of the original MAUQ to undertake further analyses and improve the use of the questionnaire.

## Abbreviations

CVI: content validity index
I-CVI: content validity index at item level
MARS: Mobile Application Rating Scale
MAUQ: mHealth App Usability Questionnaire
MAUQ-FR: mHealth App Usability Questionnaire en français
mHealth: mobile health
S-CVI: content validity index at scale level

## Data Availability

The data sets generated and analyzed during this study are available from the corresponding author upon reasonable request.
